# Beta resting-state functional connectivity predicts tactile spatial acuity

**DOI:** 10.1093/cercor/bhad221

**Published:** 2023-06-21

**Authors:** Ryoki Sasaki, Sho Kojima, Naofumi Otsuru, Hirotake Yokota, Kei Saito, Hiroshi Shirozu, Hideaki Onishi

**Affiliations:** Institute for Human Movement and Medical Sciences, Niigata University of Health and Welfare, 1398 Shimami-cho, Kita-Ku, Niigata City, Niigata 950-3198, Japan; Discipline of Physiology, School of Biomedicine, The University of Adelaide, Adelaide, South Australia 5005, Australia; Institute for Human Movement and Medical Sciences, Niigata University of Health and Welfare, 1398 Shimami-cho, Kita-Ku, Niigata City, Niigata 950-3198, Japan; Department of Physical Therapy, Niigata University of Health and Welfare, 1398 Shimami-cho, Kita-Ku, Niigata City, Niigata 950-3198, Japan; Institute for Human Movement and Medical Sciences, Niigata University of Health and Welfare, 1398 Shimami-cho, Kita-Ku, Niigata City, Niigata 950-3198, Japan; Department of Physical Therapy, Niigata University of Health and Welfare, 1398 Shimami-cho, Kita-Ku, Niigata City, Niigata 950-3198, Japan; Institute for Human Movement and Medical Sciences, Niigata University of Health and Welfare, 1398 Shimami-cho, Kita-Ku, Niigata City, Niigata 950-3198, Japan; Department of Physical Therapy, Niigata University of Health and Welfare, 1398 Shimami-cho, Kita-Ku, Niigata City, Niigata 950-3198, Japan; Institute for Human Movement and Medical Sciences, Niigata University of Health and Welfare, 1398 Shimami-cho, Kita-Ku, Niigata City, Niigata 950-3198, Japan; Department of Physical Therapy, Niigata University of Health and Welfare, 1398 Shimami-cho, Kita-Ku, Niigata City, Niigata 950-3198, Japan; Department of Functional Neurosurgery, National Hospital Organization Nishiniigata Chuo Hospital, 1-14-1 Masago, Nishi-Ku, Niigata City, Niigata 950-2085, Japan; Institute for Human Movement and Medical Sciences, Niigata University of Health and Welfare, 1398 Shimami-cho, Kita-Ku, Niigata City, Niigata 950-3198, Japan; Department of Physical Therapy, Niigata University of Health and Welfare, 1398 Shimami-cho, Kita-Ku, Niigata City, Niigata 950-3198, Japan

**Keywords:** functional connectivity, magnetoencephalography, primary somatosensory cortex, somatosensory gating, tactile spatial acuity

## Abstract

Tactile perception is a complex phenomenon that is processed by multiple cortical regions via the primary somatosensory cortex (S1). Although somatosensory gating in the S1 using paired-pulse stimulation can predict tactile performance, the functional relevance of cortico-cortical connections to tactile perception remains unclear. We investigated the mechanisms by which corticocortical and local networks predict tactile spatial acuity in 42 adults using magnetoencephalography (MEG). Resting-state MEG was recorded with the eyes open, whereas evoked responses were assessed using single- and paired-pulse electrical stimulation. Source data were used to estimate the S1-seed resting-state functional connectivity (rs-FC) in the whole brain and the evoked response in the S1. Two-point discrimination threshold was assessed using a custom-made device. The beta rs-FC revealed a negative correlation between the discrimination threshold and S1–superior parietal lobule, S1–inferior parietal lobule, and S1–superior temporal gyrus connection (all *P* < 0.049); strong connectivity was associated with better performance. Somatosensory gating of N20m was also negatively correlated with the discrimination threshold (*P* = 0.015), with weak gating associated with better performance. This is the first study to demonstrate that specific beta corticocortical networks functionally support tactile spatial acuity as well as the local inhibitory network.

## Introduction

Multiple cortical regions process tactile information in a complex manner. One of the most important cortical regions is undoubtedly the primary somatosensory cortex (S1), which mainly receives tactile inputs via the ventral posterior lateral nucleus of the thalamus (Abraira and Ginty 2013), and damage to this region causes somatosensory deficits in multiple somatosensory modalities (Kessner et al. 2016, 2019). The S1, which is located in the central area of the somatosensory system, broadly outputs somatosensory information to other regions, such as the posterior parietal cortex, secondary somatosensory cortex, primary motor cortex (M1), and insular cortex, through corticocortical connections (Jones et al. 1978; Augustine 1996; Iwamura 1998; Catani et al. 2017; Caspers and Zilles 2018). These anatomical connections imply that local processing in the S1 might support not only tactile perception but also multiprocessing between the S1 and other regions. However, it is unclear how corticocortical connections functionally contribute to the somatosensory system.

Functional connectivity (FC) has recently emerged as a key focus of neuroimaging studies assessing corticocortical networks. The term FC indicates the statistical correlation between brain signals observed in spatially separate brain regions (Brookes et al. 2012). Such interactions are considered important for serially integrating information across different regions, thus implying that correlations between brain areas are of behavioral significance (Palomar-García et al. 2017; Mizuguchi et al. 2019; Spooner et al. 2020; Arif et al. 2021). Magnetoencephalography (MEG) studies have reported corticocortical networks on M1-seed resting-state FC (rs-FC) associated with motor function, such as the M1–posterior parietal cortex, M1–superior temporal gyrus (STG), and M1–cerebellum networks (Mary et al. 2017; Sugata et al. 2020; Van Dyck et al. 2021). Regarding somatosensory function, a specific corticocortical network that contributes to the somatosensory system has not yet been identified. We previously investigated the association between cortical gray matter volume in the whole brain and 2-point discrimination (TPD) requiring tactile spatial processing to clarify which brain regions are strongly involved in the processing (Onishi et al. 2022). Importantly, our findings revealed a smaller cortical volume on the inferior parietal lobule (IPL) and middle temporal gyrus (MTG) accompanied by better TPD performance. Tactile spatial discrimination, which is crucial for recognizing the shape, size, texture, and locations of objects (Hsiao et al. 2002; Brown et al. 2004), is commonly assessed via 1- or 2-point stimuli (Kessner et al. 2016). Impairment of this ability due to stroke can lead to functional limitations in daily life (Kessner et al. 2016). However, it remains controversial whether a specific corticocortical network contributes to tactile spatial discrimination. Identification of this network could provide insights into the neural mechanisms underlying this ability and potentially contribute to the development of targeted rehabilitation strategies for individuals with impairment.

In contrast to the abovementioned lack of evidence, electroencephalography (EEG) studies have attempted to characterize the relationship between cortical response and tactile performance, focusing primarily on local responses from the S1 rather than corticocortical network and mainly assessing the first response of somatosensory evoked potentials (SEPs) using paired-pulse (PP) stimulation to elicit somatosensory gating (Höffken et al. 2007; Lenz et al. 2012). As the first response (N20/N20m) is generated from area 3b of the S1 (Allison et al. 1991; Kakigi et al. 2000), somatosensory gating enables the evaluation of inhibitory S1 function; i.e. the first stimulus attenuates the second cortical response to the second stimulus with interstimulus intervals (ISIs) of 3–300 ms (Höffken et al. 2013). Such studies have revealed a positive relationship between the inhibitory response and TPD threshold, suggesting that the local function is involved in tactile spatial acuity. However, this finding might not be generalizable because this relationship was shown only in cases of an altered rate before and after a neuroplasticity-induced intervention (Höffken et al. 2007) or in the old group (Lenz et al. 2012). Compared with EEG, MEG has additional advantages for source estimation because the magnetic fields are less affected by volume currents and anatomical inhomogeneities (Kakigi et al. 2000), allowing a more localized assessment of S1 activity. Thus, it would be notable to investigate the relationship between the inhibitory function estimated from the source level and TPD in the general population group.

Here, we used MEG to investigate whether a particular corticocortical network can predict the TPD threshold using S1-seed rs-FC in the whole brain. We also examined whether the local inhibitory network on the S1 contributes to spatial acuity. Based on our voxel-based morphometry study, we hypothesized that the strength of FC networks between S1 and IPL or MTG supports individual tactile performance as well as local inhibitory network.

## Methods

### Participants

In total, 42 healthy young adults [22 men and 20 women; age, 22.1 ± 2.2 years (mean ± standard deviation); age range, 20–32 years] participated in the current study. All participants in our previous study investigating the association between gray matter volume and TPD (Onishi et al. 2022) participated in the current study, and structure MRI and TPD performance data from that study were utilized. The Edinburgh Handedness Inventory was used to assess their handedness (Oldfield 1971), indicating that the participants tended to be right-handed [laterality score (mean ± standard deviation) = 73.2 ± 44.6]. All participants were free from neurological and psychiatric disorders, they were not taking any drugs, and they provided written informed consent before experimentation. This study conformed to the principles of the Declaration of Helsinki, and this protocol was approved by the ethics committee of Niigata University of Health and Welfare.

### Tactile spatial discrimination task and determination of the discrimination threshold

**Fig. 1:**
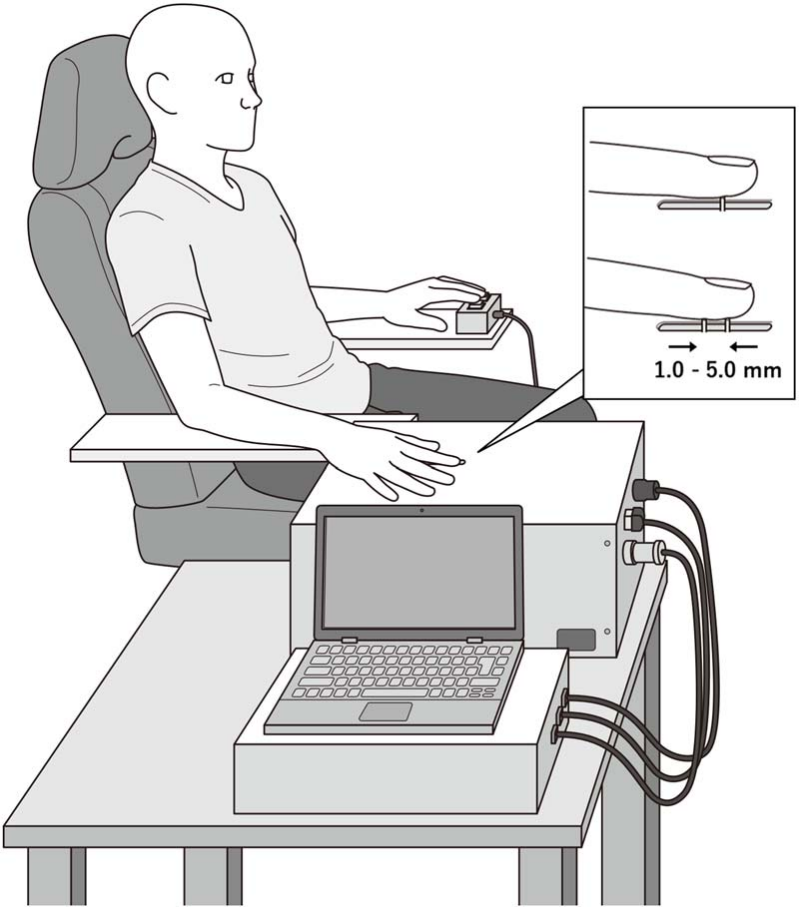
Tactile spatial discrimination task. A TPD task was conducted using the custom-made device to automatically control stimulus parameters, providing highly reliable measurements (Yokota et al. 2020). Abbreviation: TPD, 2-point discrimination.

Participants were seated with their eyes open (EO) state in a relaxed position on a comfortable chair. The TPD task was assessed using a custom-made 2-point stimulation device (Fig. 1; Takei Scientific Instruments Co. Ltd, Niigata, Japan). This motor-controlled device enables the manipulation of stimulation parameters, including pin distance (0.2–20 mm or a single pin), pin elevation speed (1–30 mm/s), stimulation depth (0.5–3.0 mm), stimulation presentation time (0.1–100 s), and stimulation interval (5–100 s) via a personal computer. In the current study, the stimulus parameters were configured as follows: pin diameter of 0.6 mm, pin elevation speed of 10 mm/s, stimulation depth of 1.0 mm, stimulation presentation time of 1 s, and stimulation interval of 5 s (Yokota et al. 2020). One or two pins were randomly presented to the tip of the right index finger at 10 different distances (0, 1.0, 1.5, 2.0, 2.5, 3.0, 3.5, 4.0, 4.5, and 5.0 mm) in 160 trials (10 distances × 16 trials). Participants responded whether they felt the sensation of 1, 2 points or were uncertain, by pressing either the left or the right button using their left hands. They pressed the right button when the stimulus was clearly identified as 2 points, whereas they pressed the left button when the stimulus was identified as 1 point or was uncertain. A binomial logistic regression model was fitted to the data to visualize the psychometric curve based on pin distances and correct answer rates. The 50% threshold was then defined as the TPD threshold (Yokota et al. 2020, 2021).

### MEG data acquisition

Participants were positioned on a custom-made nonmagnetic bed in the relaxed supine position to minimize head movements (Thibault et al. 2016), and their heads were positioned within a MEG helmet-shaped array in a magnetically shielded room (Tokin Ltd, Sendai, Japan). The position of the participant’s heads was individually adjusted to ensure appropriate contact between the helmet and their heads. A 306-channel whole-head MEG system (Elekta-Neuromag VV, MEGIN Oy, Helsinki, Finland) was used to record neuromagnetic signals sampled at 1,000 Hz with a band-pass filter (0.1–330 Hz). The 306 channels contained 102 identical triple sensors with 2 orthogonal planar gradiometers and 1 magnetometer per sensor. All participants were instructed to stay awake and not to move during the recordings.

Resting-state recording was performed in the EO state for 5 min. Electrical stimulation was then applied to the right median nerve using a bar electrode that delivered 0.2-ms square-wave constant-current pulses generated by an electrical stimulator (SEN-8203; Nihon Kohden, Tokyo, Japan) without any auditory noise. Two types of stimulation—single-pulse (SP) and PP—were randomly delivered at ISIs ranging from 3.5 to 4.5 s with the stimulus intensity set to 10% below the motor threshold that elicited a visible muscle twitch [stimulation intensity (mean ± standard deviation) = 4.6 ± 1.5 mA]. An ISI of 100 ms between pairs was used for the PP. The participants randomly received approximately 300 electrical stimulations under each condition in the EO state.

### Structural MRI processing and MEG–MRI coregistration

Structural MRI data were acquired using a 3 T Vantage Galan MRI scanner (Canon Medical Systems, Tochigi, Japan) equipped with a 32-channel head coil (QD coil, 32ch head SPEEDER, Atlas SPEEDER head/neck) and 3D T1-weighted magnetization-prepared rapid gradient echo sequence with the following parameters: inversion time, 900 ms; repetition time, 5.8 ms; echo time, 2.7 ms; flip angle, 9°; slice thickness, 1.2 mm; field of view, 23 × 23 cm^2^; scan matrix, 256 × 256; the number of slices, 160; and slice gap, no gap. The participant’s head was fixed using a head position pad. T1-weighted structural MRI data were processed using a standard pipeline in the computational anatomy toolbox (CAT12 v12.8.1; https://neuro-jena.github.io/cat/) within SPM12 (http://www.fil.ion.ucl.ac.uk/spm/software/spm12/) and MATLAB (R2019b, The Mathworks, USA). This process was initiated by applying a spatial adaptive nonlocal means denoising filter (Manjón et al. 2010), followed by the classical Markov random field approach (Rajapakse et al. 1997). The data were then bias-corrected and affine-registered, after which standard SPM unified segmentation was performed (Ashburner and Friston 2005). These preprocessed images were segmented based on the adaptive maximum a posteriori technique (Rajapakse et al. 1997) and were then refined via partial volume estimation (Tohka et al. 2004). The tissue segments were spatially normalized to the MNI template space using geodesic shooting registrations (Ashburner and Friston 2011) and were then imported into Brainstorm (v07-Jan-2022).

Prior to MEG recordings, three anatomical fiducial points of nasion and bilateral preauricular points and five indicator coils attached to participants’ heads with additional points on the head surface (approximately 200 points) were digitized using a 3D digitizer (Fastrak; Polhemus Navigator Sciences, Colchester, VT, USA). This information was used for head localization throughout the recordings. The locations of these digitized points were transformed into a standard space to coregister MEG data with individual structural MRI data. After the removal of some additional points below the nasion, an iterative closest point algorithm was used to determine a better fit between the head surface and MEG helmet (Tadel et al. 2011).

### MEG preprocessing and source modeling

The signal space separation method (Maxfilter v2.2; correction limit, 0.98; buffer length, 4 s), which efficiently separates brain signals from external signals (Taulu and Simola 2006), was performed to attenuate the environmental noise. Noise-reduced MEG data were then imported into the Brainstorm database (Tadel et al. 2011) on the MATLAB platform. Bad channels were visually inspected using the power spectrum density plot from all channels (Tadel et al. 2019). A band-pass filter of 0.5–100 Hz and notch filters of 50 Hz were used for resting-state MEG and somatosensory evoked magnetic field (SEF) data. Blink and cardiac artifacts were then removed from each continuous dataset (resting-state MEG/SEF) using an independent component analysis (ICA) via the Picard algorithm (Ablin et al. 2017). The spatial topographies of 30 components were visualized, and the components that clearly exhibited stereotyped spatial patterns consistent with blink/cardiac artifacts were removed. ICA-corrected resting-state continuous data were segmented every 2,000 ms, whereas ICA-corrected SEF data were epoched from −1,500 to 2,500 ms around the stimulus trigger for SP and first trigger for PP. The baseline was then corrected from −100 to −5 ms. Finally, all trials were visually inspected, and noisy trials including large artifacts were excluded.

After preprocessing, a head model was constructed using a forward model of overlapping spheres (Huang et al. 1999) with a constrained source model to the cortical surface. The vertices of the cortical surface were set at 15,000 points. A linearly constrained minimum variance (LCMV) beamformer (Van Veen et al. 1997; Hillebrand et al. 2005) was used to spatially filter data epochs based on data covariance, which was individually computed from resting-state (0–2,000 ms) and SEF (prestimulus period, −1,500 to −1 ms; poststimulus period, 0–2,500 ms) data in each epoch. The covariance data were individually averaged across epochs and time points for each dataset. The LCMV beamformer calculates 3D beamformer weights for each surface location and then projects them onto the cortical orientation for that location. This projection involves the multiplication of the orientation vector with the 3D weights corresponding to the source location. The LCMV regularization parameter applied to the data covariance matrix was set as its median eigenvalue to regularize the matrix, wherein eigenvalues smaller than the median eigenvalue were replaced with the median eigenvalue itself based on the tutorial (https://neuroimage.usc.edu/brainstorm/Tutorials).

### S1-seed-based FC estimations of resting-state MEG

Amplitude envelope correlation (AEC), one of the most reliable methods among a wide range of available connectivity estimation methods (Colclough et al. 2016), was used with an S1-seed-based design according to the individual N20m source (see next section). It was performed using the mean value obtained from 10 vertices (before FC computation) from 0 to 2,000 ms for the resting-state data at the source level (15,000 vertices) after signal orthogonalization to correct spurious correlations that are attributable to spatial leakage effects (Brookes et al. 2012; Hipp et al. 2012). AEC is an amplitude-based metric that evaluates the coupling between 2-time series by estimating Pearson’s correlation between the amplitude envelopes of these time series; the value is computed using the Hilbert transformation after band-pass filtering the signals (Brookes et al. 2012). In the current study, the Hilbert transformation was applied to the filtered signals of alpha (8–12 Hz) and beta (15–29 Hz) bands using the even-order linear phase finite impulse response band-pass filter. For each participant, the AEC estimated from each vertex (1 × 15,000 vertices) was averaged across epochs and time points. Rs-FC maps were individually projected onto the default anatomy (ICBM152) to obtain the grand average map and perform vertex-based analyses between the participants.

### Determination of individual source activities on the S1 and analysis of somatosensory gating

The extraction of source activities from SEF data was performed at an individual level according to previously reported procedures (Stropahl et al. 2018). Regions of interests (ROIs) were defined according to the highest activation area corresponding to individual N20m, P35m, and P60m peaks under SP and PP conditions. In the PP condition, these peaks were defined based on the first stimulus–response. To identify these ROIs, the cortical surface was smoothed, and the maximum source activity was visualized by setting the highest threshold activity. The location around the maximal activity on the S1 was regarded as the center of ROI, as determined using a “scout function,” which allows us to visually define ROIs on the brain surface (Stropahl et al. 2018). The defined activity-based scouts on the S1 had 10 vertices for each participant (Supplementary Fig. 1).

After the construction of aligned individual SP and PP source maps consisting of absolute values of current amplitude (Tadel et al. 2019), the SP source map was subtracted from the PP source map to remove the first SEF waveform under the PP condition. This approach removes the interfering waveform induced by the first pulse, assuming that the combined response under the PP condition can be linearly separated into distinct components (Höffken et al. 2013). Following this approach, the subtracted waveform was converted into absolute values for display purpose. The values were represented by time shifting (−100 ms) to correspond to the stimulus trigger under SP condition and were then baseline-corrected from −100 ms to −5 ms under both conditions.

At the ROI level, the individual peak amplitude and latency were analyzed in the range of 20–26, 27–40, and 45–71 ms corresponding to individual ROIs (N20m, P35m, and P60m; Supplementary Fig. 2) in each condition from the absolute SP maps and subtracted absolute PP source maps. Somatosensory gating on each peak was eventually expressed as a ratio of the data for PP and SP conditions.

To obtain grand average source maps of each SEF component across participants, the source maps were individually projected onto the default anatomy (ICBM152), and spatial smoothing of the source maps was then applied using a 3-mm full-width half maximum value (Tadel et al. 2019). These source maps were subsequently averaged across participants and finally averaged across time points (20–25, 30–40, and 50–70 ms) for display purpose.

### Statistics

All analyses were performed using SPM12 (Wellcome Trust Center for Neuroimaging, https://www.fil.ion.ucl.ac.uk/spm/software/spm12/) or PASW statistics software version 28 (SPSS; IBM, Armonk, NY, USA).

Rs-FC maps were imported into SPM12 to perform vertex-based analysis. A regression analysis (2-tailed) of the alpha or beta rs-FC map was performed to investigate the relationship between the S1-seed rs-FC map and tactile performance. Multiple comparisons were subsequently conducted across all vertices using threshold-free cluster enhancement (TFCE; E = 0.5, H = 2; 10,000 permutations) to avoid selecting arbitrary cluster-forming thresholds (Smith and Nichols 2009). Further, TFCE maps were assessed using a cluster-wise threshold of *P*_FWE-corr_ < 0.05 and a cluster-forming threshold of *k* > 50 vertices. In the regression analysis, if surviving clusters were detected at the threshold, the location was identified based on gyral-based neuroanatomical regions using the Desikan–Killiany atlas (Desikan et al. 2006). The location of the peak vertex was also identified in each brain region in significant clusters (*P*_FWE-corr_ < 0.05), followed by 2-tailed Spearman’s rank correlation tests between rs-FC at each peak vertex and TPD for visualization of the effect of the direction. For SEFs, Wilcoxon signed-rank test with Bonferroni correction was used for amplitudes between stimulus conditions (*α* = 0.05/3). Two-tailed Spearman’s rank correlation tests with Bonferroni correction were performed between the gating ratios (N20m, P35m, and P60m) and tactile performance (*α* = 0.05/3). To confirm a relationship between corticocortical and local networks showing significant relationships with tactile performance (see Result section), 2-tailed Spearman’s rank correlation tests with Bonferroni correction were also performed between N20m gating and beta rs-FC in the left superior parietal lobule (SPL), IPL, and STG extracted from peak vertices (*α* = 0.05/3). Unless otherwise stated, all data are presented as the mean ± standard error of the mean.

## Results

All 42 participants completed MEG and tactile performance procedures. The TPD threshold was set to 2.58 ± 0.08 mm (Onishi et al. 2022). No bad channels were detected from the power spectral density plots; however, some trials were excluded (resting-state MEG: 2.5% ± 0.4%; SEF_SP: 3.3% ± 0.3%; and SEF_PP: 3.4% ± 0.3%). ICA removed some components that were consistent with blink and cardiac artifacts from each dataset (resting-state MEG: electrooculogram (EOG) = 1.7 ± 0.1, electrocardiogram (ECG) = 1.1 ± 0.1; and SEF:EOG = 2.0 ± 0.1, ECG, 1.1 ± 0.1).

### Rs-FC

**Fig. 2:**
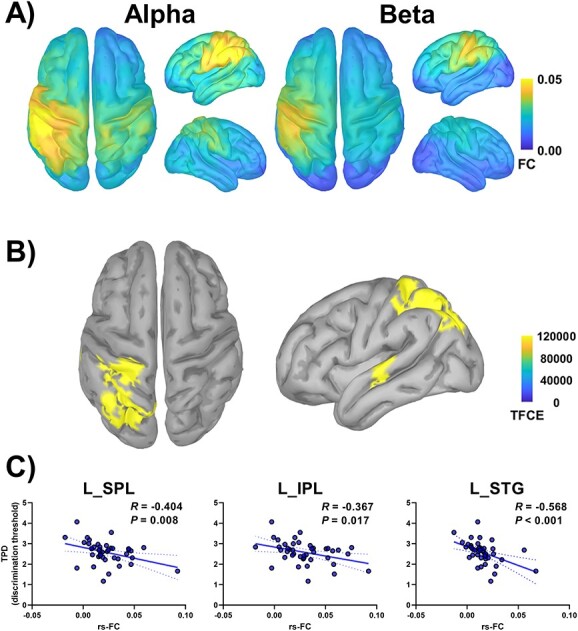
Grand average S1-seed rs-FC maps and relationship between the beta rs-FC and TPD. (A) The source maps show grand average S1-seed rs-FC at the alpha (*left panel*) and beta (*right panel*) bands. (B) The source map shows a significant relationship between the beta rs-FC and TPD within the colored vertices (*P*_FWE-corr_ < 0.05) in the left SPL, IPL, and STG, as assessed using the Desikan–Killiany atlas. (C) Significant relationships are observed between the beta rs-FC extracted from the peak vertex in each area and TPD. The solid blue lines represent trend lines, whereas the dashed blue lines represent the 95% confidence interval. Abbreviations: L_IPL, left inferior parietal lobule; rs-FC, resting-state functional connectivity; L_SPL, left superior parietal lobule; L_STG, left superior temporal gyrus; S1, primary somatosensory cortex; TFCE, threshold-free cluster enhancement; TPD, 2-point discrimination.

Grand average S1-seed rs-FC maps are shown in Figure 2A. A regression analysis revealed that clusters were identified between beta rs-FC values and TPD in the left SPL, IPL, and STG (Fig. 2B and Supplementary Fig. 3; cluster 1: *k*_E_ = 484, *P*_FWE-corr_ < 0.048; cluster 2: *k_E_* = 52, *P*_FWE-corr_ < 0.049). For the peak vertex in each region, Spearman’s rank correlation tests revealed negative relationships between the beta rs-FC and TPD (SPL: *R* = −0.404, *P* = 0.008; IPL, *R* = −0.367, *P* = 0.017; STG, *R* = −0.568, *P* < 0.001) (Fig. 2C); strong FC was associated with a lower discrimination threshold (better performance). No clusters were observed for other comparisons.

### SEF and somatosensory gating

**Fig. 3:**
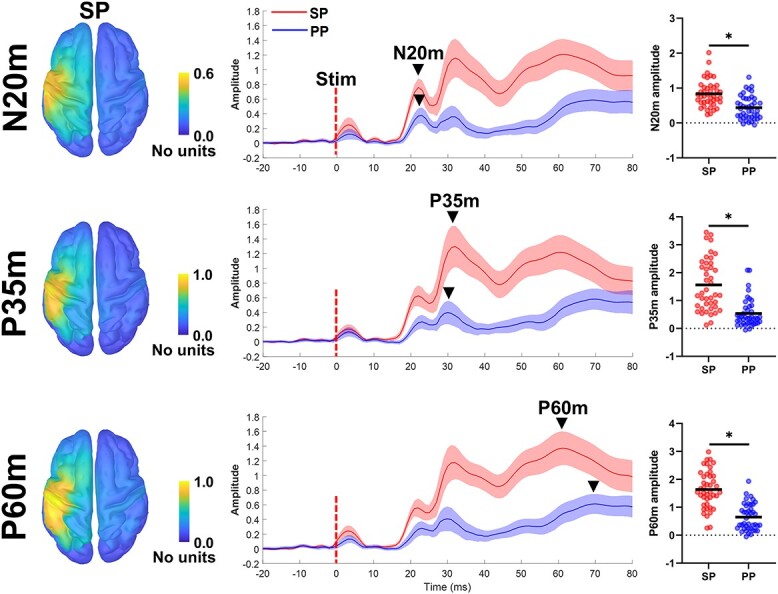
Grand average source maps and SEF waveforms. (*Left panel*): Grand average source maps on N20m, P35m, and P60m in the SP condition. (*Middle panel*): Grand average SEF waveform extracted from individual ROIs of N20m, P35m, and P60m in the SP and PP conditions. The red dashed line represents the timing of electrical stimulation, whereas the black rectangle represents the SEF peak extracted from each source. The red (SP) and blue (PP) lines represent the mean source activity across participants, whereas these shaded areas represent 95% confidence intervals. (*Right panel*): Comparisons of cortical activation between conditions with mean and individual values. Abbreviations: PP, paired-pulse; SP, single-pulse; stim, stimulation.

**Fig. 4:**
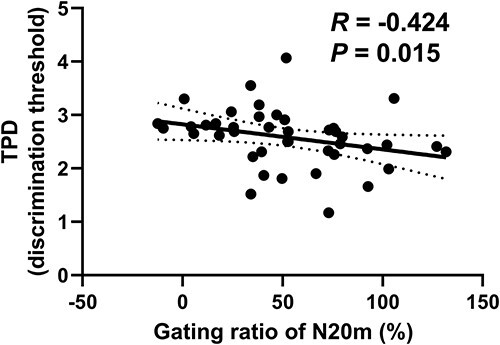
Relationship between the gating ratio of N20m and TPD. Unbroken line represents the trend line, whereas dashed lines represent the 95% confidence interval. Abbreviation: TPD, 2-point discrimination.

The grand average source maps obtained under SP condition clearly showed activations around the left S1 following right median nerve stimulation (Fig. 3). The grand average SEF waveform obtained from individual ROIs also showed clear peaks (Fig. 3; mean latency: SP_N20m, 22.4 ± 0.2 ms; PP_N20m, 22.7 ± 0.2 ms; SP_P35m, 32.7 ± 0.6 ms; PP_P35m, 32.0 ± 0.5 ms; SP_P60m, 58.3 ± 1.2 ms; and PP_P60m, 60.4 ± 1.5 ms). After the subtraction and conversion into absolute values under the PP condition, some participants slightly showed negative peak amplitudes due to the influences of strong inhibition and baseline correction. These activations varied between conditions, with smaller responses observed for the PP condition (Fig. 3; all *P* < 0.001). Correlation analysis revealed that the gating ratio of N20m was negatively correlated with TPD (Fig. 4; *R* = −0.424, *P* = 0.015); however, no other correlations were detected (all *P* > 0.85).

### Relationship between somatosensory gating of N20m and rs-FC

No correlations were found between somatosensory gating of N20m and the beta S1-seed rs-FC in the left SPL, IPL, and STG (all *P* > 0.58).

## Discussion

Using MEG at the source level, we aimed to investigate the mechanism by which corticocortical and local networks contribute to tactile spatial acuity, as assessed using a computer-controlled 2-point stimulator. Importantly, the beta S1-seed rs-FC was negatively correlated with the TPD threshold in the left SPL, IPL, and STG. We then characterized the relationship between local responses and the TPD threshold, revealing that the inhibitory response in N20m was negatively correlated with spatial acuity.

## Beta rs-FC on spatial acuity

In this study, we applied the AEC metric to rs-FC estimations from various FC methods because the metric can efficiently remove signal leakage using a symmetric orthogonalization procedure (Brookes et al. 2012; Hipp et al. 2012), indicating one of the most consistent methods in rs-FC metrics (Colclough et al. 2016). Using the AEC metric in the whole brain, the current study identified relationships between S1-seed rs-FC and TPD in the left SPL, IPL, and STG; strong beta connectivity facilitated TPD performance, whereas weak connectivity disturbed TPD performance. Importantly, these brain regions are in a similar agreement with those from our previous voxel-based morphometry study, which revealed correlations with TPD; IPL and MTG were associated with TPD [SPL and angular gyrus (AG) also tended to be correlated (uncorrected *P* < 0.05)] (Onishi et al. 2022). These findings indicate that the posterior parietal cortex, except for the supramarginal gyrus (SMG), is involved in tactile spatial processing. Gray matter volume is generally considered to reflect various factors, such as cell density, cell size, synaptic density, myelin sheath, glia cell size and number, capillaries, water content, and GABA_A_ receptor concentration (Morrison and Hof 1997; Draganski et al. 2004; Zatorre et al. 2012; Pomares et al. 2017), neural oscillations are thought to be synchronized patterns of synaptic activity (Buzsáki and Draguhn 2004). Therefore, it is plausible to suggest that variations in gray matter volume among individuals could have a linear impact on synaptic activity originating from the gray matter. This could explain the similarities observed between the current and our previous studies.

### Functional connection of regions involved in tactile spatial processing

In general, the posterior parietal cortex consists of 2 main regions, SPL, which is located in the posterior part of the posterior parietal cortex, and IPL, which includes the inferior part of the posterior parietal cortex (Caspers and Zilles 2018). Notably, the Desikan-Killiany atlas separates the IPL and SMG into distinct subregions but does not define a specific region for the AG (Desikan et al. 2006) (Supplementary Fig. 3). Studies have shown that both SPL and IPL have strong anatomical connections with the postcentral gyrus in the human and monkey brains (Catani et al. 2017). As a functional role, SPL integrates different sources of information from somatosensory, visual, and motor areas for high-order cognitive processes (Passarelli et al. 2021). In the somatosensory process, lesions in the SPL can cause tactile agnosia, which is an inability to recognize objects through touch (Kubota et al. 2017), suggesting that the SPL contributes to the perception of objects by touch (Passarelli et al. 2021). IPL including the AG but not the SMG also mediates various high-order cognitive processes, such as attention, spatial cognition, reading, comprehension, and memory retrieval (Seghier 2013); however, this region has not been well studied anatomically and functionally because its location varies between studies and its sole activation by tasks is challenging (Seghier 2013). Nevertheless, considering these functional roles and the process required during TPD (i.e. judgment of 1- or 2-point stimuli), SPL and IPL might be involved in the integration of tactile spatial processing with the S1. In contrast to these regions, STG is not typically considered as a primary contributor to the somatosensory system as it is associated with auditory processing and language comprehension (Bhaya-Grossman and Chang 2022). However, there is evidence that the temporal lobe may play a role in integrating tactile information with other sensory modalities, particularly in the context of social communication (Blakemore et al. 2005; Gordon et al. 2013). Furthermore, previous works suggest that the temporal lobe integrates multisensory information (Schroeder et al. 2001; Beauchamp et al. 2004). Beauchamp et al. (2004) reported that both auditory and visual stimuli activate the superior temporal sulcus. Schroeder et al. (2001) showed that the auditory association cortex is responsive to both auditory and somatosensory stimuli, suggesting that the temporal lobe and S1 are functionally connected to integrate multisensory information. At the anatomical level, this region is abundantly connected to SPL and IPL via the middle longitudinal fascicle in humans (Makris et al. 2013). As mentioned earlier, our previous study revealed a correlation between TPD and gray matter volume in the temporal lobe (around the MTG) (Onishi et al. 2022). Overall, this evidence potentially supports our findings regarding the association between FC in S1–STG and tactile performance. However, the functional role of the somatosensory system remains unclear. Therefore, further investigation is warranted to determine whether STG is involved in tactile spatial processing.

### Local brain oscillations on spatial acuity

Whereas alpha oscillations closely couple to tactile perception in the S1 (Baumgarten et al. 2016; Brickwedde et al. 2019), beta oscillations are known to be associated with movement in the M1 (Espenhahn et al. 2019, 2020). Despite this fact, the beta-synchronized network specifically contributed to tactile performance. The association with beta oscillations is usually reported by correlating a motor task with beta power in a local area (i.e. M1) (Espenhahn et al. 2019, 2020; Xifra-Porxas et al. 2019) but not in corticocortical networks. In the current study, time–frequency transformation was supplementarily employed to investigate the relationship between local oscillatory responses [i.e. event-related desynchronization (ERD) or rebound synchronization (ERS)] and tactile spatial acuity (Supplementary methods). In particular, beta oscillations in the sensorimotor cortex are known to reflect GABAergic activity. For example, post-movement beta ERD is facilitated by the GABA agonist diazepam, whereas beta rebound ERS remains unaffected by the drug (Hall et al. 2011). Tiagabine, a blocker of the GABA transporter that enhances endogenous GABA activity, facilitates post-movement beta ERD while reducing beta rebound ERS (Muthukumaraswamy et al. 2013). Furthermore, post-movement or post-stimulus beta rebound ERS is positively correlated with GABA concentration (Gaetz et al. 2011; Cheng et al. 2017). This accumulating evidence suggests that beta changes in S1 are specifically associated with tactile spatial acuity as well as somatosensory gating. Despite this, we could not determine any correlations between local oscillatory responses and TPD performance (Supplementary results and Fig. 4), suggesting that the mechanisms of inhibitory networks are distinct between local beta changes and somatosensory gating in tactile spatial processing. Altogether, beta oscillations might play a more important role in corticocortical networks in terms of tactile spatial processing.

### Somatosensory gating on spatial acuity

S1 responses have often been evaluated using SEPs/SEFs; in particular, the first response has been applied as an index of S1 excitability. These responses, including N20m, P35m, and P60m, reflect different processes in the S1 (Huttunen et al. 2006). N20m reflects excitatory postsynaptic potentials projecting onto area 3b from the thalamus, P35m reflects inhibitory postsynaptic potentials in area 3b, and P60m may exhibit multiple activations involving both postsynaptic potentials in area 1 or 2 (Huttunen et al. 2006, 2008). Somatosensory gating is a robust inhibitory phenomenon that has often been assessed in studies on neurophysiological mechanisms, and it is commonly considered to represent the “filtering” of redundant stimulus features at an early level of processing (Hsiao et al. 2013; Cheng et al. 2015). Pharmacological studies have attempted to identify this inhibitory mechanism, which was believed to be involved in GABAergic circuits, using a GABA_A_ agonist; however, this drug failed to modify somatosensory gating on N20/N20m (Huttunen et al. 2008; Stude et al. 2016). Although the exact inhibitory mechanisms remain unknown, our findings reveal a negative correlation between somatosensory gating on the S1 and the discrimination threshold. This is supported by our earlier study, which showed that a decrease in somatosensory gating after non-invasive brain stimulation to the S1 led to improved somatosensory function (Sasaki et al. 2022). Although our study primarily focused on investigating the relationship between somatosensory gating and tactile spatial discrimination, it is conceivable that tactile temporal discrimination is more related to somatosensory gating. Nonetheless, we decided to assess tactile spatial discrimination as the primary measure due to its widespread use in clinical research settings (Kessner et al. 2016), potentially yielding broad applicable findings. One possible explanation for the association between somatosensory gating and tactile spatial discrimination is that the S1 may be involved in processing both temporal and spatial information. This hypothesis is supported by previous studies, which have shown that high-frequency tactile stimulation facilitates not only tactile temporal discrimination (Erro et al. 2016) but also tactile spatial discrimination (Kalisch et al. 2010; David et al. 2015; Erro et al. 2016). However, further research is warranted to elucidate the specific mechanisms underlying this shared activity and its contribution to the observed association between the temporal inhibitory network and tactile spatial acuity.

Conversely, Lenz et al. (2012) reported that somatosensory gating was positively correlated with TPD. However, different population groups or analytical methods might explain the contradictions observed between these studies; Lenz et al. (2012) only assessed the correlation in the older population group (mean age, 70.2 ± 6.2 years) and used an ISI of 30 ms, whereas the current study used an ISI of 100 ms in the young population group. Furthermore, Lenz et al. (2012) assessed somatosensory gating of N20/P25 amplitude using EEG, whereas we assessed N20m amplitude using MEG. N20/P25 amplitude might reflect multiple processing stages between areas 3b and 1 (Allison et al. 1991), whereas N20m amplitude only represents the activation of area 3b (Kakigi et al. 2000). These findings suggest that the 2 conflicting studies evaluated different aspects of somatosensory processing.

### Bottom-up and top-down processing

Considering the advantage of source estimation in MEG, the current study provides an important implication that the inhibitory function on the S1 negatively influences tactile spatial processing in the young population group. Although local and corticocortical networks were correlated with tactile spatial processing, these roles might be distinct in the somatosensory system because no correlations were observed between these networks. In general, early-latency SEF is believed to reflect upcoming somatosensory information from the body in a “bottom-up” manner (Wiesman and Wilson 2020). In contrast, the beta rs-FC is likely to reflect the integration of tactile spatial processing in a “top-down” manner as the posterior parietal cortex is involved in high-order cognitive function (Seghier 2013; Passarelli et al. 2021).

## Conclusion

In conclusion, we identified functional corticocortical networks contributing to tactile spatial acuity, which was associated with the left S1–SPL, –IPL, and –STG at the beta-band. Notably, the identified regions were in a similar agreement with those of our previous voxel-based morphometry study (Onishi et al. 2022). These findings indicate that multiple networks coordinately contribute to tactile spatial processing. Furthermore, we found a negative relationship between TPD and inhibitory response in the S1, suggesting that abundant local inhibitory activity can disturb upcoming information, leading to decreased tactile spatial acuity. Overall, S1-seed beta corticocortical networks as well as the local network are involved in tactile spatial acuity.

## Supplementary Material

Supplementary_Data_bhad221

## Data Availability

The neuroimaging data could not be made openly available because of ethical restrictions. Access to and reuse of data require a data-sharing agreement between institutions as well as approval from the requesting researcher’s local ethics committee. If needed, please contact the corresponding author, Dr Ryoki Sasaki (hwd17005@nuhw.ac.jp).
