# Supplementary material for: Beta resting-state functional connectivity predicts tactile spatial acuity
Source: Cereb Cortex. 2023 Jun 21;33(16):9514–23. doi: 10.1093/cercor/bhad221 (PMC10431746; doi:10.1093/cercor/bhad221)
Supplement: Supplementary_Data_bhad221 [file supplementary_data_bhad221.zip › Supplementary_Data_bhad221.docx]

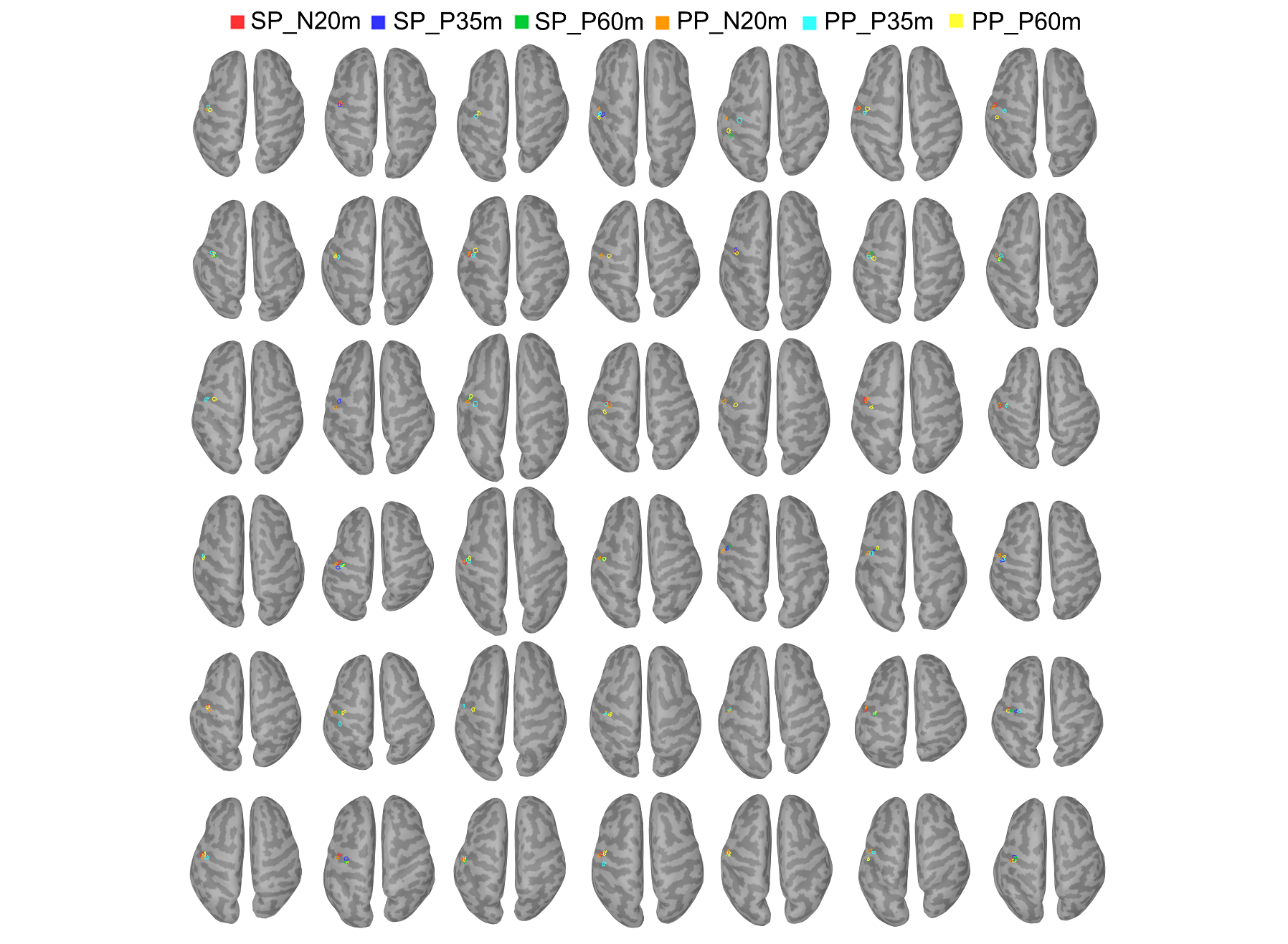


**Supplementary Figure 1.** **Localization of ROIs.** Location of each SEF peak (i.e., N20m, P35m, and P60m) based on SP and PP conditions at the individual level. If all colors do not appear on a whole brain map, it indicates that some ROIs share a location. Abbreviations: PP, paired-pulse; ROI, region of interest; SEF, somatosensory-evoked magnetic field; SP, single-pulse.


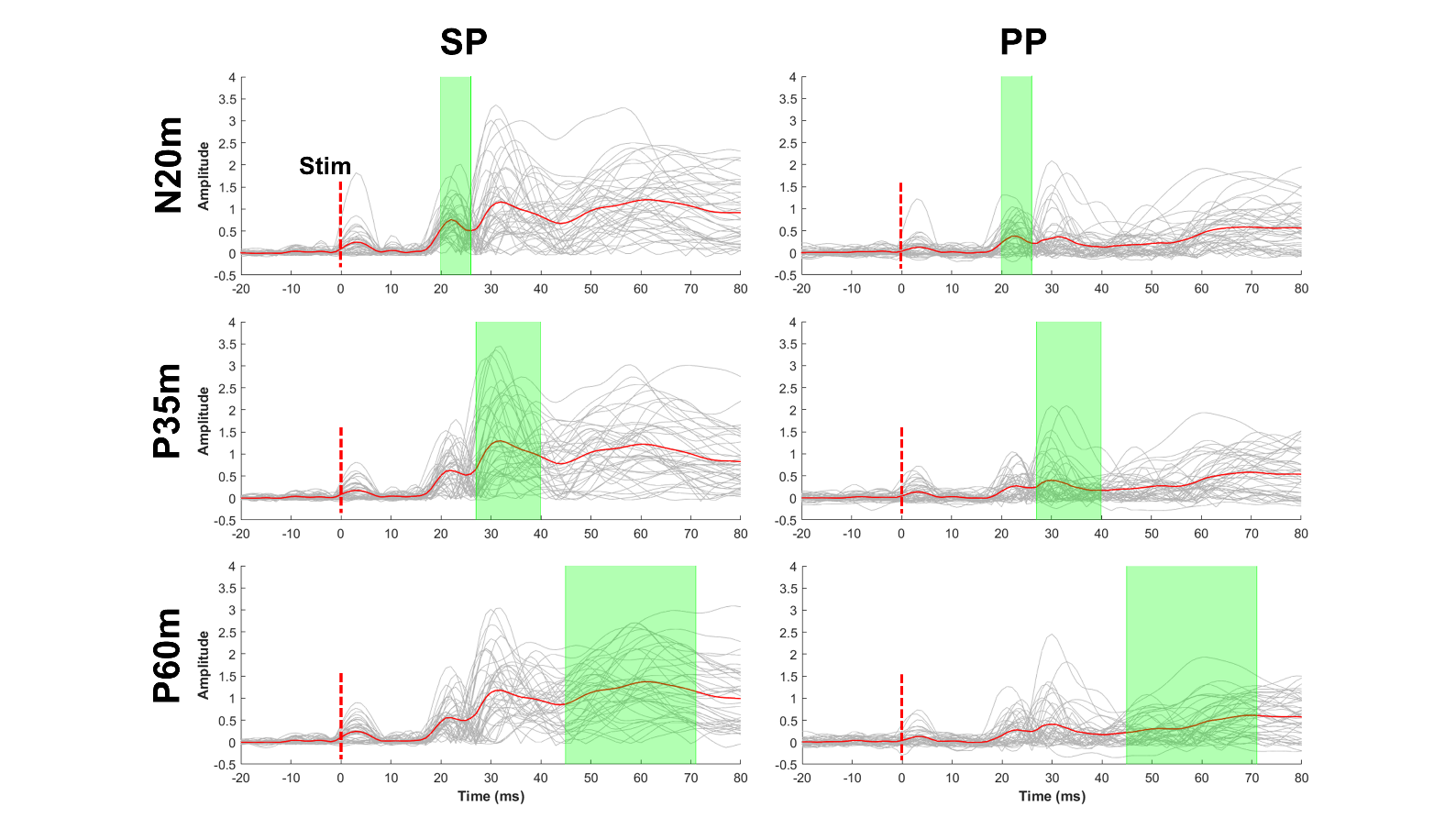


**Supplementary Figure 2.** **Individual responses induced by median nerve stimulation in the ROIs.** The red line represents average waveform across participants, and the gray line represents individual waveforms extracted from individual ROIs of N20m, P35m, and P60m in the SP and PP conditions. The green area shows the range of the individual peaks of N20m, P35m, and P60m. Abbreviations: PP, paired-pulse; ROI, region of interest; SP, single-pulse; stim, stimulation.


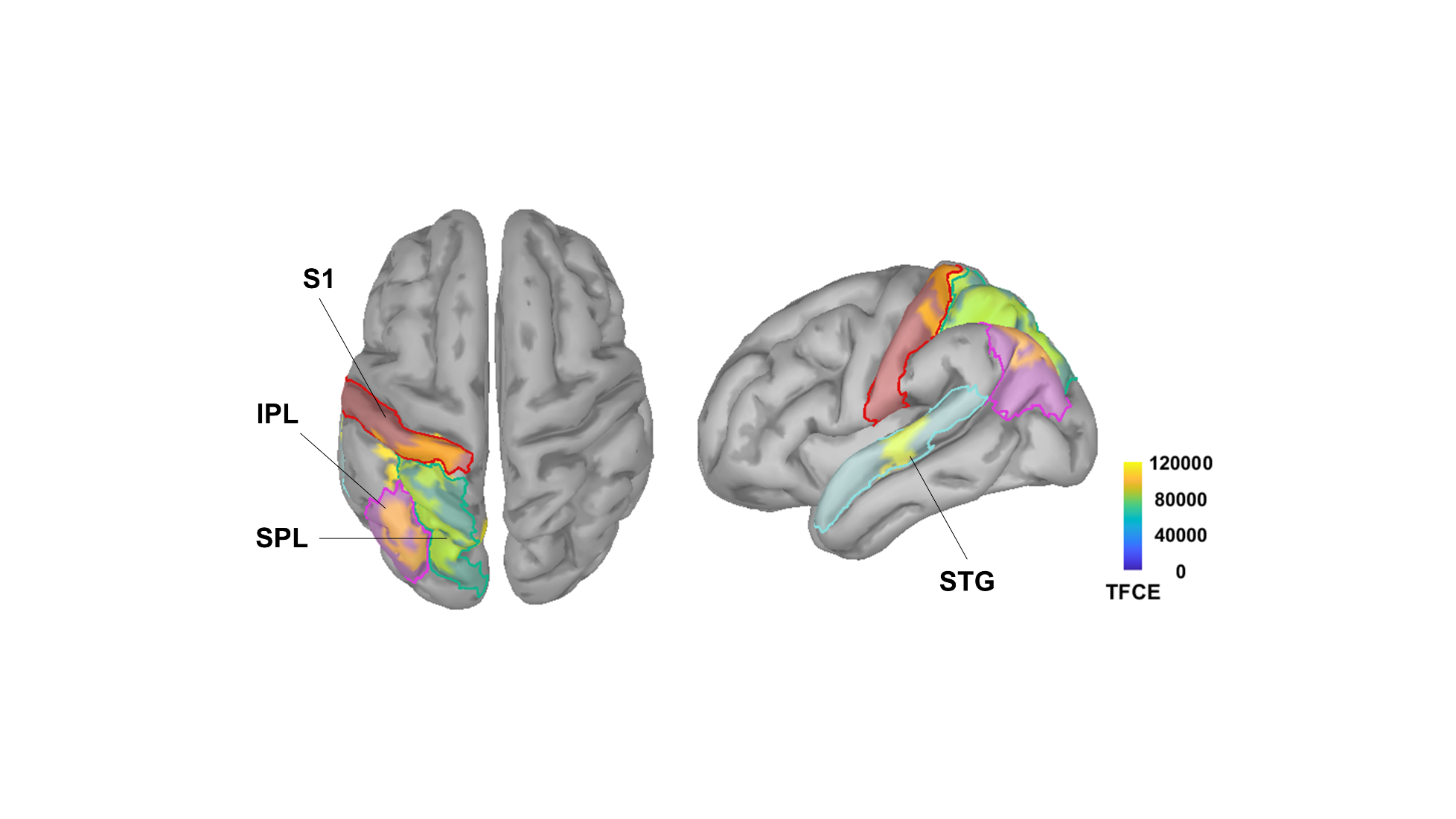


**Supplementary Figure 3.** **Identification of brain areas forming significant clusters.** The location of significant clusters was identified based on the Desikan–Killiany atlas. These clusters were primarily located in the left SPL, IPL, and STG. Abbreviations: IPL, inferior parietal lobule; SPL, superior parietal lobule; STG, superior temporal gyrus; S1, primary somatosensory cortex; TFCE, threshold-free cluster enhancement.

***Supplementary methods for time-frequency transformation on SEFs***

Complex Morlet wavelets ranging from 5 to 100 Hz were applied to the estimated individual source area of N20m with the mother wavelet (central frequency = 1 Hz; full-width half-maximum = 3 s) in each trial of the SP condition. The time-frequency (TF) data were then averaged across epochs and normalized by baseline correction from −1000 to −50 ms using the dB scale (Gyurkovics et al. 2021). Oscillation changes occurring in specific periods, such as event-related desynchronization (ERD; 98−692 ms) and synchronization (rebound ERS; 396−1382 ms) were identified, and the peak amplitudes were analyzed in the alpha and beta bands. Two-tailed Spearman’s rank correlation tests with Bonferroni correction were performed to compare these responses with TPD (α = 0.05/4).

***Supplementary results for time-frequency transformation on SEFs***

Grand average TF spectra obtained from the source of N20m showed a temporal decrease and increase in power (i.e., ERD and rebound ERS, respectively) at these bands (alpha ERD = 381.2 ± 14.4 ms; alpha rebound ERS = 892.2 ± 30.7 ms; beta ERD = 188.4 ± 7.5; beta rebound ERS = 594.4 ± 19.2 ms; Supplementary Fig. 4A). Individual plots are also displayed in Supplementary Fig. 4B and C. No correlations were detected between each response and tactile performance (all *P* > 0.54).


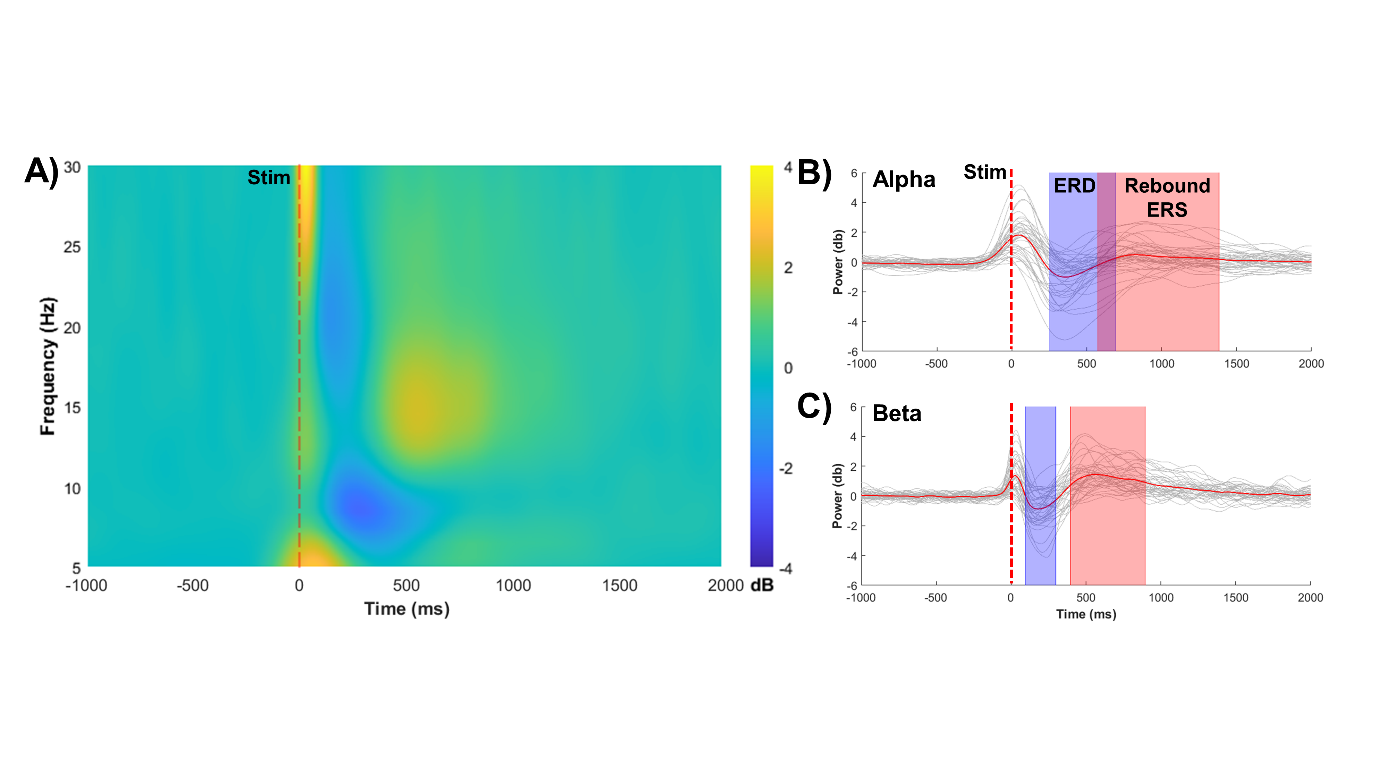


**Supplementary Figure 4.** **Oscillation changes in the S1 following median nerve stimulation.** (*A*) Grand average spectrograms extracted from the source of N20m in the SP condition. (*B, C*) Individual waveforms of brain oscillatory activity under the SP condition at each oscillation band. Red lines represent the means of individual waveforms, whereas gray lines represent individual waveforms. The blue area shows the range of individual peaks of ERD, and the red area shows the range of individual peaks of rebound ERS. Red dashed lines represent the timing of electrical stimulation. Abbreviations: ERD, event-related desynchronization; ERS, event-related synchronization; S1, primary somatosensory cortex; SP, single-pulse; Stim, stimulation.

**Reference**

Gyurkovics M, Clements GM, Low KA, Fabiani M, Gratton G. The impact of 1/f activity and baseline correction on the results and interpretation of time-frequency analyses of EEG/MEG data: A cautionary tale. Neuroimage. 2021:237:118192.
